# Synthesis of Copper Nanoparticles Stabilized with Organic Ligands and Their Antimicrobial Properties

**DOI:** 10.3390/polym13172846

**Published:** 2021-08-25

**Authors:** Noemi Jardón-Maximino, Marissa Pérez-Alvarez, Gregorio Cadenas-Pliego, Luis E. Lugo-Uribe, Christian Cabello-Alvarado, José M. Mata-Padilla, Enrique Díaz Barriga-Castro

**Affiliations:** 1 Centro de Investigación en Química Aplicada (CIQA), Saltillo 25294, Coahuila, Mexico; jardonmaximino@gmail.com (N.J.-M.); christian.cabello@ciqa.edu.mx (C.C.-A.); jose.mata@ciqa.edu.mx (J.M.M.-P.); enriquediaz.barriga@ciqa.edu.mx (E.D.B.-C.); 2 Centro de Tecnología Avanzada CIATEQ, Lerma 52004, Estado de México, Mexico; luis.lugo@ciateq.mx; 3 CONACYT-Centro de Investigación y de Innovación del Estado de Tlaxcala, Tlaxcala C.P. 90000, Tlaxcala, Mexico

**Keywords:** chemical reduction method, copper nanoparticles, polyethylenimine, zeta potential, antimicrobial

## Abstract

In this work, we report the synthesis of copper nanoparticles (Cu NPs), employing the chemical reduction method in an aqueous medium. We used copper sulfate pentahydrate (CuSO_4_·5H_2_O) as a metallic precursor; polyethylenimine (PEI), allylamine (AAM), and 4-aminobutyric acid (AABT) as stabilizing agents; and hydrated hydrazine as a reducing agent. The characterization of the obtained nanoparticles consisted of X-ray, TEM, FTIR, and TGA analyses. Through these techniques, it was possible to detect the presence of the used stabilizing agents on the surface of the NPs. Finally, a zeta potential analysis was performed to differentiate the stability of the nanoparticles with a different type of stabilizing agent, from which it was determined that the most stable nanoparticles were the Cu NPs synthesized in the presence of the PEI/AAM mixture. The antimicrobial activity of Cu/PEI/AABT toward *P. aeruginosa* and *S. aureus* bacteria was high, inhibiting both bacteria with low contact times and copper concentrations of 50–200 ppm. The synthesis method allowed us to obtain Cu NPs free of oxides, stable to oxidation, and with high yields. The newly functionalized Cu NPs are potential candidates for antimicrobial applications.

## 1. Introduction

Copper has been identified as a material with excellent antimicrobial properties, as it can efficiently eliminate fungi, bacteria, and viruses [1]. The antimicrobial activity of copper was demonstrated with the emergence of the COVID-19 pandemic, since copper was able to inactivate the novel coronavirus (SARS-CoV-2) in shorter times compared to other materials [2]. 

Regarding Cu NPs, several papers have recently highlighted their importance in priority areas for humankind, for instance, in medical (the COVID-19 pandemic) [3,4], farming [5,6,7,8], and environmental applications [9,10], among others. As with other metallic nanoparticles (M NPs), Cu NPs have been widely studied due to their antimicrobial, electrical, thermal conductivity, catalytic, and optical properties [11].

The development of polymeric composites is an important application of M NPs [12]. Some material properties, such as electrical, mechanical, thermal, and antimicrobial properties, have been improved by the incorporation of M NPs into polymeric matrices [13,14,15]. Nevertheless, there are important difficulties to obtaining M NPs polymeric composites with a suitable dispersion due to the strong tendency of the M NPs to agglomerate, as well as their incompatibility with the polymeric matrix [14,15]. An effective strategy employed to overcome these problems involves the functionalization of M NPs with organic ligands or polymers [12].

The attractiveness of Cu NPs is mainly due to their higher abundance and lower cost compared to gold and silver nanoparticles [1,16]. An important disadvantage of Cu NPs is that they are easily oxidized. However, the oxidation problem can be avoided using stabilizing agents, such as organic ligands or polymers, during the synthesis of the nanoparticles [17].

Several methods have been developed to synthesize Cu NPs, with the chemical reduction and green synthesis methods being the most studied. Green synthesis is a simple and low-cost method which leads to stable products. The green synthesis method is environmentally friendly, since the chemical reduction agents are replaced by nontoxic natural compounds [16]. An example of this green route is the synthesis of Cu NPs in the presence of cotton without the addition of chemical reducing agents, which exclusively produces oxidation-stable Cu NPs in a facile manner [18]. On the other hand, the method of chemical reduction in an aqueous medium is one of the most widely used for Cu NPs synthesis due to the water’s capability to dissolve a wide variety of metallic salts and the method’s reproducibility [19,20]. Using this method, it is possible to control the size, morphology, and composition of Cu NPs by varying the experimental conditions, such as the concentration of reagents, temperature, solvents, metal precursor, reducing agent, and stabilizing agents that prevent the aggregation of nanoparticles, as well as the nature of the dispersing medium [21,22,23].

The effect of the experimental conditions on the synthesis of Cu NPs has been studied with the chemical reduction method. Copper ions can be reduced to Cu, Cu_2_O, or CuO depending on the reducing power of the reducing agent [22]. Some reports have suggested that pH values in the reaction medium between 9 and 10.5 have an influence on obtaining pure Cu NPs. A mixture of Cu and Cu_2_O nanoparticles was achieved at pH values as high as 12. At low pH values, the formation of CuO and Cu_2_O is prevented [22,23,24,25]. In addition, by varying the concentration of the stabilizing agent, the size and shape of the nanoparticles can be controlled [23]. Wang and coworkers reported a synthesis route in an aqueous medium to produce Cu NPs with a controlled size and composition, employing copper (II) salt, hydrazine, and poly (acrylic acid) (PAA). The authors controlled the Cu NPs size by varying the PAA concentration, obtaining an average particle size (APS) ranging from 30 to 80 nm [24]. Lai and coworkers synthesized Cu NPs in an aqueous medium using CuSO_4_·5H_2_O, polyvinylpyrrolidone (PVP), and sodium hypophosphite. The authors observed that, with a higher PVP concentration, the overgrowth of the Cu NPS was prevented [22]. Wang and Asefa reported the synthesis of Cu NPs of uniform size using CuSO_4_·5H_2_O, hydrazine, poly(allylamine) (PAAm), and NaOH to control the solution’s pH. PAAm had an important effect on the long-term stability of the Cu NPs [23]. The production of Cu NPs composed exclusively of Cu(0), free of CuO and Cu_2_O, increased the number of applications, presented a higher effectiveness in antimicrobial activity, and were less cytotoxic compared to CuO nanoparticles [26]. Recently, our research group reported the synthesis of Cu NPs by chemical reduction in an aqueous solution and constant basic pH, using nitrogen-based ligands as stabilizing agents. With our method, the production of CuO and Cu_2_O depended on the type and amount of the ligand utilized during the synthesis process. The Cu NPs coated with nitrogen-based ligands were stable to oxidation under ambient conditions for long-term periods after being synthesized, as they did not undergo oxidation after 3–5 years of storage. The nitrogen-based ligand content in the Cu NPs ranged from 1.3 wt.% to 5.1 wt.% [20,27,28]. 

Currently, polyethylenimine (PEI), a highly branched cationic polymer, is of great interest in the synthesis of M NPs in aqueous media [29]. PEI acts as a reactive template in which amino groups coordinate with metal ions dissolved in water, reducing the aggregation capability of the M NPs due to the steric hindrance provided by the PEI [30,31,32]. Pulkkinen and coworkers compared PEI and tetraethylenepentamine (TEPA) in the synthesis of Cu NPs. They observed that the higher amino groups’ content of PEI promoted the formation of smaller Cu NPs. The average particle size of the nanoparticles was 8.5 nm for PEI and 19.4 nm for TEPA [17]. A similar effect was reported by Bowen Wang and coworkers. The authors synthesized Cu NPs in the presence of PEI, observing that the Cu NPs size was reduced with an increment in PEI concentration. This behavior was attributed to an anchorage of the Cu NPs to the PEI polymeric chain, reducing the aggregation capability of the Cu NPs [11].

In this study, Cu NPs were synthesized by the chemical reduction method in an aqueous medium using CuSO_4_·5H_2_O as the metal precursor, hydrazine as a reduction agent, and NaOH as a pH stabilizer in the reaction medium. Several stabilizing agents were evaluated, such as polyethylenimine (PEI), a blend of polyethylenimine and allylamine (PEI/AAM), and a blend of polyethylenimine and 4-aminobutyric acid (PEI/AABT). 

In addition, a study regarding antimicrobial activity toward *Pseudomonas aeruginosa* and *Staphylococcus aureus* bacteria was carried out. The current interest in exploring metal NPs for antimicrobial applications is linked to the growing microbial resistance to multiple antimicrobial agents and the development of resistant strains [33]. A common practice to analyze bacterial protection comprises an evaluation of antimicrobial activity toward Gram-positive and Gram-negative bacteria, since the resistance of both types of bacteria to traditional antibiotics is a matter of concern in the health area. Some examples of Gram-positive bacteria are *Staphylococcus aureus* and *Enterococcus*, while examples of Gram-negative include *Escherichia coli, Klebsiella pneumoniae, Pseudomonas aeruginosa*, and the *Acinetobacter* species [34].

## 2. Materials and Methods

### 2.1. Materials

The synthesis of Cu NPs was carried out using branched polyethylenimine (PEI), Mn ~ 1800 by GPC, Mw ~ 2000 by light scattering (Ls), 50 wt.% in H_2_O, allylamine (AAM) ACS reagent ≥ 98%, 4-aminobutyric acid (AABT) ACS reagent ≥ 99%, sodium hydroxide (NaOH) ACS reagent ≥ 97%, and copper (II) sulfate pentahydrate (CuSO_4_·5H_2_O) ACS reagent ≥ 98%. All chemicals were supplied by Sigma Aldrich (St. Louis, MO, USA) and were used as received without further treatment.

### 2.2. Synthesis of Cu NPs

In a 500 mL ball flask partially immersed in an oil bath at 60 °C, 1.0 g of CuSO_4_·5H_2_O was dissolved in 66 mL of deionized water with mechanical stirring. Then, the stabilizing agents (1.4 g of AABT or 10.3 g of AAM), dissolved in 20 mL of water, were added. The flask was kept in agitation for 10 min. Then, 0.123 g of PEI dissolved in 20 mL of H_2_O was added, followed by the slow addition of 60 mL of 0.5 M NaOH. The mixture was left to react for 30 min, and then 4.8 mL of hydrazine was added to obtain a solution pH of 12. The reaction was maintained for 60 min at 60 °C with constant stirring. Then, the reaction mixture was allowed to reach room temperature, and the Cu NPs were quickly purified to avoid oxidation.

The Cu NPs separation from the solution was performed by centrifugation at 13,000× rpm for 20 min at room temperature, followed by two wash steps with acetone and centrifugation under the same conditions. Finally, the Cu NPs were dried in a vacuum oven at 70 °C for 5 h. 

### 2.3. Characterization Techniques

X-ray diffraction (XRD): For this test, a Siemens D-5000 diffractometer (Aubrey, TX, USA) with a scanning interval in the 20 scale from 20° to 80° and a scan speed of 0.02% was used. Copper Kα radiation was employed with a wavelength of 1.54 Å. Values of 25 mA and 35 kV were used for intensity and voltage, respectively.

Thermogravimetric analysis (TGA) (TA instruments Inc., New Castle, PA, USA): A DuPont Instruments 951 analyzer was utilized. The operating conditions consisted of a heating rate of 10 °C/min and an air atmosphere with a gas flow of 50 mL/min. The samples run were performed from 30 °C to 600 °C in an N_2_ atmosphere. Once 600 °C was reached, the N_2_ atmosphere was switched to O_2_ for a better combustion of organic components.

Fourier-transform infrared spectroscopy (FTIR) (PerkinElmer Inc., Waltham, MA, USA): FTIR was used to obtain the FTIR spectra. Cu NPs were blended with moisture-free potassium bromide (KBr), and a thin disk was prepared with this mixture. The disks were analyzed in the spectral range from 400 cm^−1^ to 4000 cm^−1^. This analysis was used to identify the presence of the organic coatings on the nanoparticles surface. 

Zeta potential: The zeta potential analysis was carried out in a Zetasizer Nano ZS equipment from Malvern Instruments Ltd., Malvern, UK. The Cu NPs were previously dispersed for 5 min with an ultrasound, using distillated water as dispersion medium. 

### 2.4. Antibacterial Activity 

The antibacterial activity of Cu-PEI/AABT NPs sample was determined according to methods reported in ASTM E2149-01. *Pseudomonas aeruginosa* (ATCC 13388) Gram-negative and *Staphylococcus aureus* (ATCC 6538) Gram-Positive were used for the tests, they were purchased from American Type Culture Collection (Manassas, VA, USA). The procedure to evaluate the antibacterial activity was recently reported by our research group [35].

The antibacterial activity of the composites was calculated using Equation (1) [36], where *C_o_* is the number of formed CFUs (colony-forming units) in the control dish (bacterial solution without sample), and *C* is the number of formed CFUs in the dishes where the bacterial solution was in contact with the sample.

(1) $$\mathrm{Antibacterial}\;\mathrm{activity}\;\left(\%\right)=\frac{C_{o}\;-\;C}{C_{o}}\text{×}100$$

## 3. Results and Discussion

### 3.1. X-ray Diffraction Analysis of Cu NPs 

**Figure 1:**
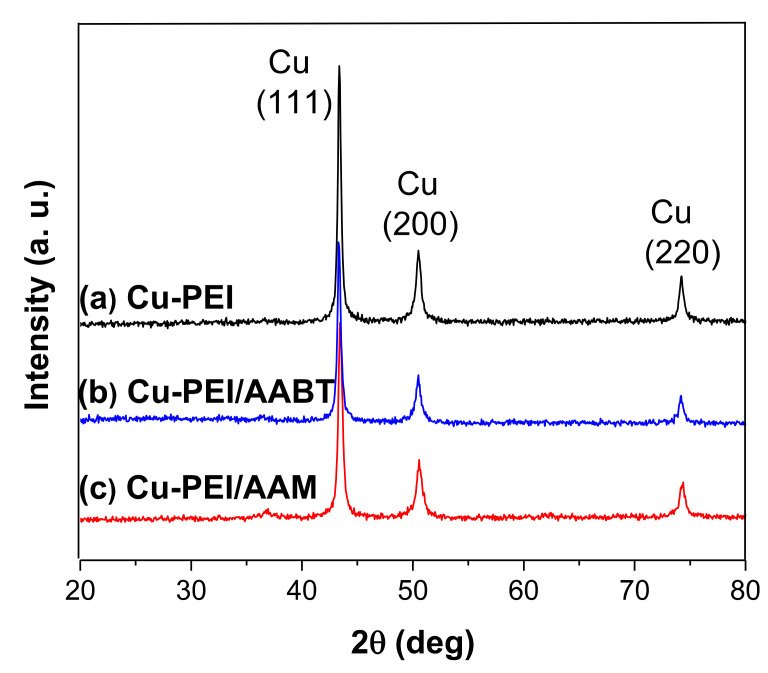
XRD diffractograms of Cu NPs synthesized with different stabilizers. (**a**) Cu-PEI NPs, (**b**) Cu-PEI/AABT NPs, and (**c**) Cu-PEI/AAM NPs.

Figure 1 shows the X-ray diffractograms of the Cu NPs synthesized with different stabilizers, identified as (a) PEI, (b) PEI/AABT, and (c) PEI/AAM. In all samples, the composition of the nanoparticles was mostly metallic Cu. The diffraction peaks at the 2θ scale at 43°, 50°, and 74° correspond, respectively, to crystallographic planes (111), (200), and (220), which are the principal diffraction angles for Cu reported by the International Centre for Diffraction Data JCDPS [37,38,39]. The synthesis of Cu NPs was performed at a constant pH of 12.0, a condition that favors the formation of CuO and Cu_2_O. However, no oxidized copper species were detected. This suggests that the nitrogen-based ligands are effective in preventing the oxidation of Cu NPs during synthesis.

The Debye–Scherrer equation (Equation (2)) [40] was applied to calculate the average particle diameter using the mean width of the diffraction peak located at 2θ of 43.3° for Cu:

D = Kλ/βcosθ(2)

where D is the particle diameter, K is the Scherrer constant (0.89), λ is the radiation wavelength (Cu = 1.54056 Å), β is the mean width of the most intense diffraction peak (in radians), and θ is the Bragg diffraction angle. The diameter of the PEI-, PEI/AABT-, and PEI/AAM-stabilized particles showed an average particle size of 24.3 nm, 26.6 nm, and 19.3 nm, respectively. These results suggest that a higher content of the stabilizer (see Section 3.2) decreases the size of the nanoparticles, as reported by other authors [11]. 

### 3.2. Thermogravimetric Analysis (TGA) 

**Figure 2:**
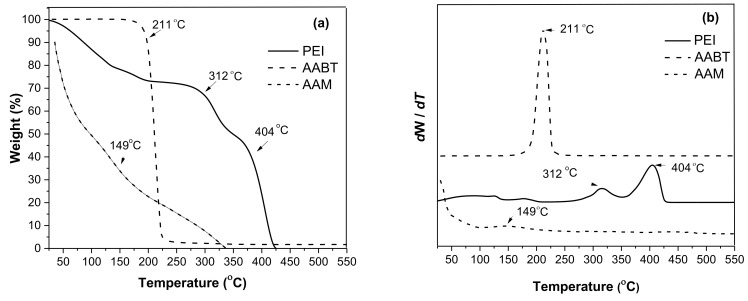
TGA thermograms of the stabilizers (**a**) and DTG curves of the stabilizers (**b**).

Figure 2 shows the weight loss percentage (2a) and DTG (2b) curves obtained from the TGA analysis of the stabilizers. PEI showed a weight loss of 17% around 100 °C, followed by a stable weight plateau, and then a strong degradation between 300 °C and 425 °C. The DTG curve of PEI (Figure 2b) shows two important degradation peaks at 312 °C and 404 °C. With AABT, thermal degradation occurred in a single step at 211 °C, as shown in the DTG curve of AABT. AAM exhibited a weight loss from the beginning of heating, showing a degradation peak at 149 °C in the DTG curve. The TGA analysis revealed that the low molecular weight ligands were less thermally stable than the PEI polymeric ligands.

**Figure 3:**
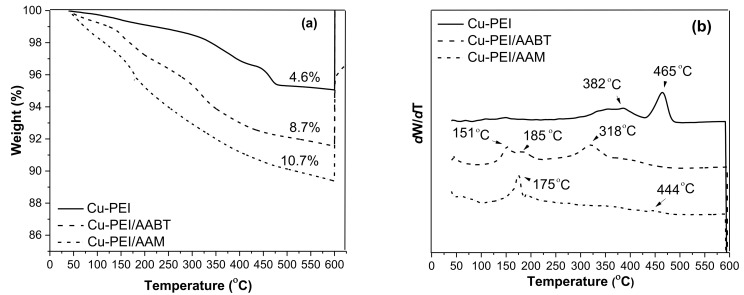
TGA thermograms of the Cu NPs with different ligands (**a**) and DTG curves of the Cu NPs with different ligands (**b**).

The weight loss curves corresponding to the Cu NPs samples synthesized with the PEI, PEI/AABT, and PEI/AAM stabilizers are shown in Figure 3a. The organic coating content was estimated from the residual weight value at 600 °C, obtaining a value of 4.6% for PEI, 8.7% for PEI/AABT, and 10.7% for PEI/AAM. All samples showed an increase in thermal decomposition as the temperature increased, but the behavior of the weight loss curves was different than that of the stabilizers alone. Upon reaching 600 °C, O_2_ was supplied for better combustion of the organic material. This caused the oxidation of the copper, which resulted in a weight gain at 600 °C in all samples.

The DTG curves, presented in Figure 3b, also show different behaviors between the curves corresponding to the Cu NPs coated with the ligands and the curves corresponding to the organic ligands. The decomposition peak temperatures of the Cu-PEI NPs, Cu-PEI/AABT NPs, and Cu-PEI/AAM NPs samples (Figure 3b) were higher than the temperatures obtained for the stabilizers (Figure 2b). This behavior suggests a strong interaction between the copper and the polar groups of the stabilizers. Such interaction could be an advantage in producing polymeric nanocomposites [35], but could also have negative effects on other applications, such as electrically conductive materials, where it is desirable to remove the coating from Cu NPs at low temperatures [17]. At 600 °C, complete thermal degradation of the organic material and weight gain as a result of oxidation of the metallic copper was observed. 

The reaction yield calculation for the synthesis of Cu NPs and the particle diameter were obtained according to procedures reported previously [20], and the results are shown in Table 1. As mentioned above, there was a relationship between the ligand content and the average particle size. In all cases, the particle size was relatively small, similar to those obtained in other studies using the chemical reduction method. 

It is interesting to compare these results with others reported in the literature. For instance, Cadenas-Pliego and coworkers used allylamine (AAm) and polyallylamine (PAAm) for the synthesis of Cu NPs, reporting an average particle size and ligand wt.% similar to the values presented in Table 1 [20]. Pulkkinen and coworkers obtained an average particle size of 8.5 nm and a higher ligand wt.% using PEI (Mw = 1200). The difference in the results can be attributed to the reaction conditions employed and the use of PEI with a low molecular weight [17].

### 3.3. Transmission Electron Microscopy Analysis (TEM)

**Figure 4:**
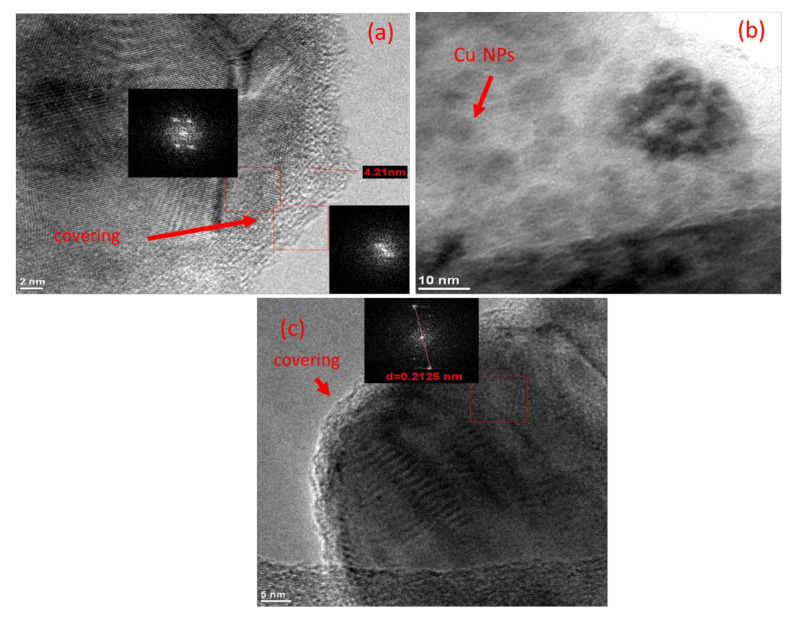
TEM micrographs of (**a**) Cu-PEI NPs, (**b**) Cu-PEI/AABT NPs, and (**c**) Cu-PEI/AAM NPs.

The morphology of Cu NPs was determined by transmission electron microscopy analysis (TEM). Figure 4a shows the micrograph of the synthesized nanoparticles in the presence of PEI, which presents a spheric morphology. The micrograph obtained at a high resolution indicates that the Cu NPs had a coating (PEI) on their surface with an estimated thickness of 4.2 nm. Figure 4b shows the micrograph of the nanoparticles synthesized in the presence of the PEI/AABT blend. Single spherical particles were observed, as well as some aggregates. The nanoparticles synthesized in the presence of the PEI/AAM blend also presented a spherical morphology. The micrographs obtained with a high resolution also provide evidence of the coating on the Cu NPs (Figure 4c).

### 3.4. Fourier Transform Infrared Spectroscopy Analysis (FTIR)

Through FTIR analysis, it was possible to determine the presence of PEI, AAM, and AABT on the surface of the Cu NPs. To determine whether the copper was coordinated with the amino groups of the stabilizers, we analyzed whether there was a shift in the absorption bands corresponding to the C–N or N–H bond. A shift change suggests the interaction of nitrogen with the copper atom, as proposed as an explanation for similar systems [41].

**Figure 5:**
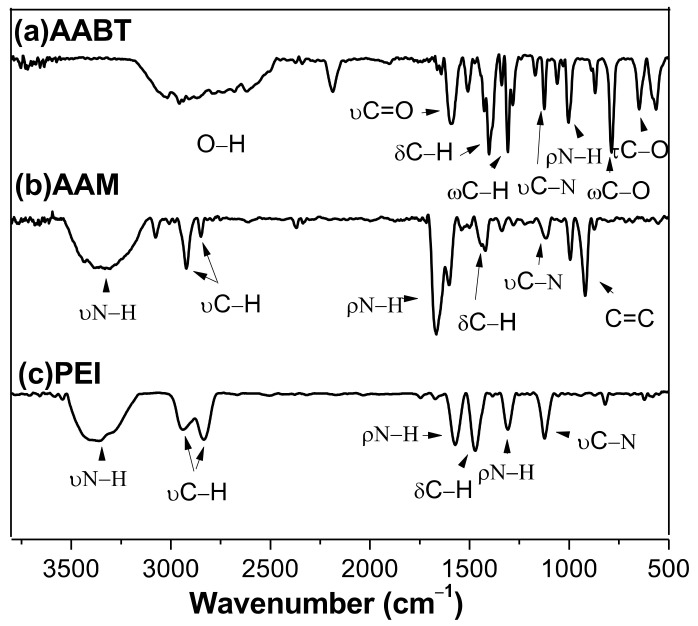
FTIR spectra of (**a**) AABT, (**b**) AAM, and (**c**) PEI.

The FTIR spectra of the pure PEI, AAM, and AABT stabilizers are illustrated in Figure 5, and the characteristics absorption bands are shown in Table 2.

**Figure 6:**
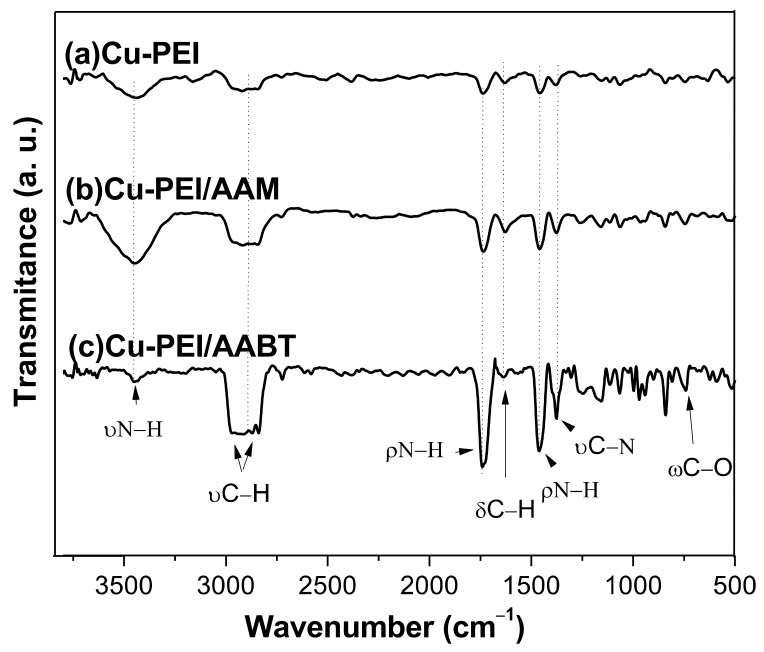
FTIR spectra of the Cu NPs with different stabilizers, (**a**) Cu-PEI; (**b**) Cu-PEI/AAM; (**c**) Cu-PEI/AABT.

Figure 6 presents the FTIR spectra of the Cu-PEI NPs, Cu-PEI/AAM NPs, and Cu-PEI/AABT NPs samples. The spectra include the characteristic bands of the pure PEI with some differences in the wavelength values. The N-H bond bending signal at 1310 cm^−1^ and the C-N bond stretching signal at 1120 cm^−1^ were slightly wider and changed in their displacement compared to pure PEI signals. These changes can be explained by the coordination of copper to the free electron pair of the nitrogen atom. The presence of AAM is difficult to visualize because it has the same -NH_2_ functional group as PEI, and the band corresponding to the C=C group was not observed. This can be attributed to two factors: (1) a low AAM content in the sample, and (2) a possible polymerization reaction. A similar explanation was reported for Cu NPs synthesized in the presence of a blend of polyallylamine/allylamine [27]. With the Cu NPs synthesized in the presence of the blend PEI/AABT, the most characteristic signal that suggests the existence of AABT is the corresponding to C=O group at 1600 cm^−1^. Unfortunately, this band overlapped with the N-H band of the PEI. However, the bending signals in the wagging and twisting mode of the C-O bond can be observed [42,43,44,45]. 

### 3.5. Zeta Potential Analysis of Cu NPs

**Figure 7:**
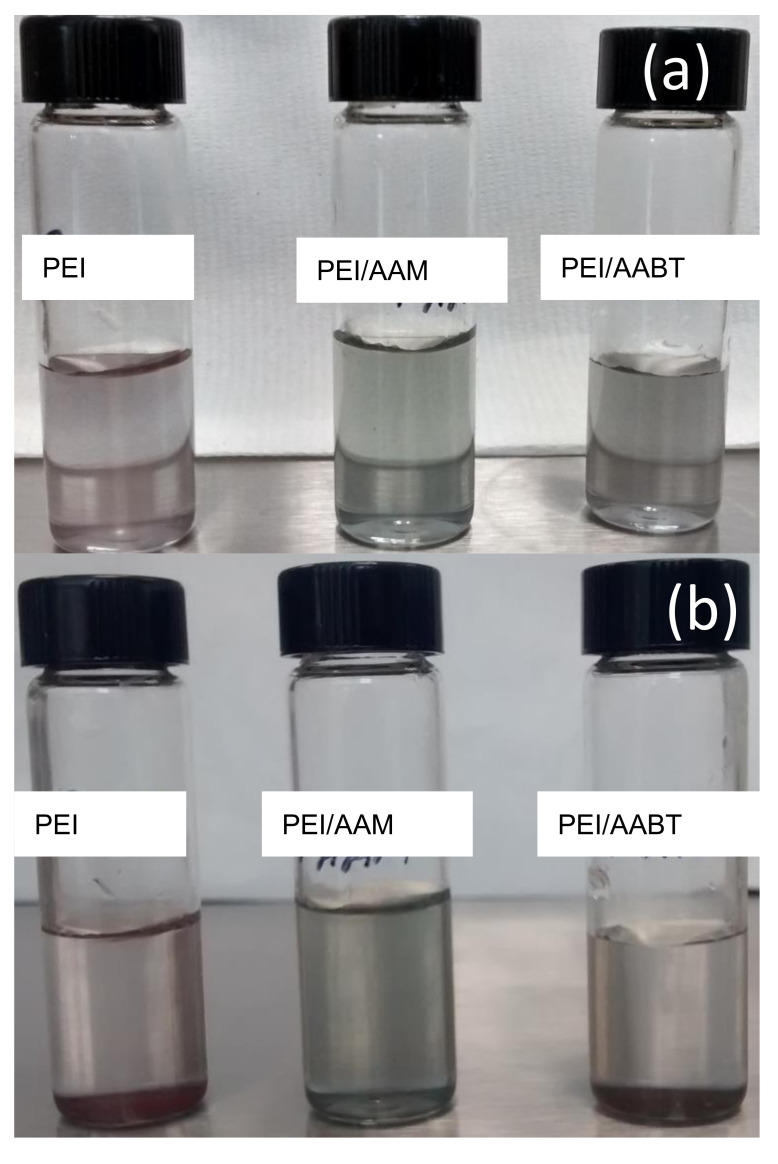
Colloidal stability of the Cu NPs (**a**) after 10 h of dispersion, and (**b**) after 24 h of dispersion.

The Cu-PEI NPs, Cu-PEI/AAM NPs, and Cu-PEI/AABT NPs samples were analyzed by zeta potential analysis. The comparison of the results provides a measure of the dispersion capability of the Cu NPs with different stabilizers. The samples were dispersed in distilled water by ultrasound, and the zeta potential was immediately measured. The obtained values were −68.1 mV, −82.8 mV, and −68.6 mV, respectively. All samples had negative values lower than −30 mV. This result suggests good colloidal stability of the Cu NPs and can be attributed to the high coating content (see Section 3.2). The highest negative value was achieved by the Cu PEI/AAM NPs sample, which also had the highest organic coating content (10.7%). All solutions maintained good colloidal stability, as there are no signs of precipitation after 10 h. For all solutions, signs of precipitation were only observed after 24 h. Figure 7 shows pictures of the solutions at 10 and 24 h after the dispersion of the Cu NPs. 

### 3.6. Antibacterial Activity of Cu-PEI/AABT NPs

The Cu-PEI/AABT NPs samples were selected for evaluation in an antibacterial activity test, since these nanoparticles obtained the highest yield and showed a good dispersion in water after ultrasonication. The minimum bactericidal concentration (MBC), which was also obtained in this study, is defined as the lowest concentration of an antimicrobial agent that kills 100% of the microorganisms.

**Figure 8:**
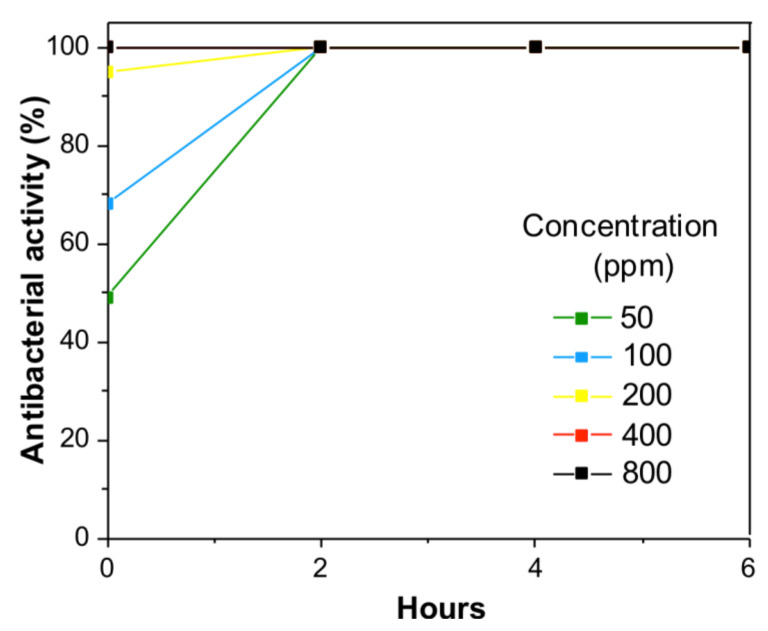
Percentage of the antibacterial activity of the Cu-PEI/AABT NPs against *P. aeruginosa* at different concentrations.

**Figure 9:**
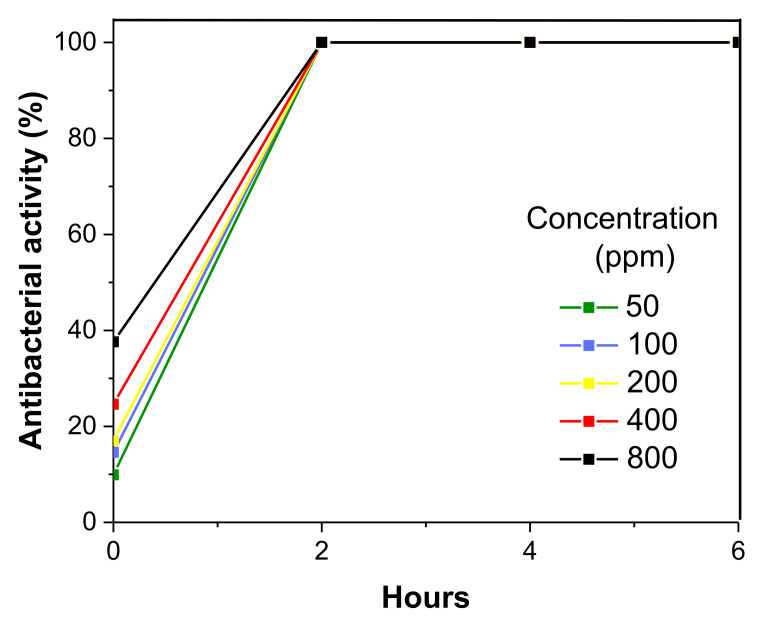
Percentage of the antibacterial activity of the Cu-PEI/AABT NPs against *S. aureus* at different concentrations.

The antibacterial activity of the Cu NPs was determined toward *P. aeruginosa* and *S. aureus* bacteria using Cu NPs concentrations of 50 ppm, 100 ppm, 200 ppm, 400 ppm, and 800 ppm with contact times between 0 and 6 h. Figure 8 and Figure 9 show the results for both bacteria.

The antibacterial activity plots show that the Cu NPs effectively inhibited the growth of both bacteria from the beginning of the contact time. It can be observed that the antibacterial activity increased with a higher Cu NPs concentration. *P. aeruginosa* bacteria were the most sensitive to Cu NPs (Figure 8). At zero contact time for the concentration of 50 ppm, 100 ppm, 200 ppm, 400 ppm, and 800 ppm, the Cu NPs showed an antibacterial activity percentage of 48.9%, 68.2%, 94.9%, and 100%, respectively. Thus, from the first contact time, the CU NPs killed 100% of *P. aeruginosa* bacteria at a minimum bactericidal concentration (MBC) of 200 ppm. The Cu NPs can behave as antimicrobial agents from this concentration.

*S. aureus* bacteria was the most resistant to the Cu NPs. Even at the highest concentration of 800 ppm, only 40% inhibition was achieved at zero contact time (Figure 9). The behavior observed in Figure 9 suggests that the antibacterial activity of 99.9% was achieved at a contact time of 2 h for all the Cu NPs concentrations. Based on these results, we can confirm that the MBC of these Cu NPs was 50 ppm at 2 h of contact time. These data are comparable to those reported for Cu NPs with high antimicrobial activity [33]. Moreover, the antimicrobial activity of the Cu NPs reported in this study is also comparable with commercial nanoparticles, since it has been reported that Cu NPs with 99.8% purity and an average particle size of 25 nm show an MBC toward *Pseudomonas aeruginosa* and *Staphylococcus aureus* between 800 ppm and 1600 ppm [14]. Cu NPs with high antimicrobial activity are potential candidates for applications in polymeric membranes for water purification [1]. 

## 4. Conclusions

The Cu NPs synthesis using the organic stabilizers polyethylenimine, allylamine, and 4-aminobutyric acid led to the obtention of oxide-free Cu NPs with high yields, small average particle size, and the ability to form stable colloidal solutions. The presence of the organic coating was confirmed by the FTIR, TEM, and TGA analyses. The colloidal stability observed in the Cu NPs can be attributed to the organic coating formed on their surfaces. The colloidal solution obtained with Cu-PEI/AAM NPs showed the highest stability with a zeta potential of −82.8 mV. The antibacterial activity of Cu-PEI/AABT toward *P. aeruginosa* and *S. aureus* bacteria was significantly high. Cu-PEI/AABT can inhibit both bacteria with low contact times at copper concentrations between 50–200 ppm. The dispersion effortlessness of Cu NPs and their high antibacterial activity can play an important role in antimicrobial applications. Such applications may involve the direct spraying on textiles or the synthesis of polymeric nanocomposites.

## Figures and Tables

**Table 1:** Cu NPs synthesis results.

Ligand	Cu ^a^ (%)	Yield (%)	Ligand ^a^ (%)	Average Particle Size ^b^ (nm)
PEI	95.4	89.6	4.6	24.3 ± 0.4
PEI/AABT	91.3	90.9	8.7	26.6 ± 0.4
PEI/AAM	89.3	86.3	10.7	19.3 ± 0.4
PAAm ^c^	96.8	86.0	3.2	24.0 ± 0.4
AAM ^c^	94.4	36.0	5.6	30.0 ± 0.4
PEI ^d^	87.5	-	12.5	8.5

^a^ determined by TGA, ^b^ determined by XRD, ^c^ Ref. [20], ^d^ Ref. [17].

**Table 2:** FTIR spectral data (cm^−1^) of PEI, AAM, and AABT [42,43,44,45,46,47,48,49,50,51,52].

Assignment	PEI	AAM	AABT
Stretching N−H	3360	3370	3380
Stretching C–H	2950 ^a^	2920 ^a^	2960
	2830 ^s^	2840 ^s^	2940
Bending N–H	1570	1670	1010
	1310		
Bending C–H	1480	1420	1400
			1310
Stretching C–N	1120	1120	1120
Stretching C=O			1600
C=C		920	
Bending C–O			789
			648

^a^ asymmetrical, ^s^ symetrical.

## Data Availability

The data presented in this study are available on request from the corresponding author.
